# HIV prevalence and sexual behavior among young male conscripts in the Brazilian army, 2016

**DOI:** 10.1097/MD.0000000000009014

**Published:** 2018-05-25

**Authors:** Rosa Dea Sperhacke, Leonardo Rapone da Motta, Sérgio Kakuta Kato, Andréa Cristina Vanni, Machline Paim Paganella, Maria Cristina Pimenta de Oliveira, Gerson Fernando Mendes Pereira, Adele Schwartz Benzaken

**Affiliations:** a Laboratório de Pesquisa em HIV/AIDS (LPHA), Área do Conhecimento de Ciências da Vida, Universidade de Caxias do Sul (UCS), Caxias do Sul; b Programa de Pós Graduação em Ciências da Reabilitação, Universidade Federal de Ciências da Saúde de Porto Alegre, Porto Alegre, RS; c Departamento de Vigilância, Prevenção e Controle das Infecções Sexualmente Transmissíveis, do HIV/Aids e das Hepatites Virais, Secretaria de Vigilância em Saúde, Ministério da Saúde, Brasília, DF, Brazil.

**Keywords:** army, conscripts, HIV, prevalence, sexual behavior, young men, young MSM

## Abstract

Since 1996, the Brazilian Department of Sexually Transmitted Infections (STI), AIDS and Viral Hepatitis (Departamento de Vigilância, Prevenção e Controle das IST, do HIV, AIDS e Hepatites Virais, Secretaria de Vigilância em Saúde, Ministério da Saúde) in collaboration with the Brazilian Ministry of Defense has conducted periodic and anonymous probability sample surveys to determine the human immunodeficiency virus (HIV) prevalence, the sexual and risk behaviors among conscripts of the Brazilian army. This study aimed to estimate the HIV seroprevalence of conscripts in Brazil by geographic region and to describe behavior in relation to the risk of HIV transmission by analyzing data from the Brazilian Conscripts Survey 8th edition performed in 2016.

Conscripts were selected with a 2-stage sampling method stratified by geographical region. The study included a self-reported questionnaire and blood collection for HIV, hepatitis, and syphilis testing. Data from 37,282 conscripts between 17 and 22 years of age were analyzed. Of these conscripts, 73.7% stated that they were sexually active. The overall country-wide prevalence of HIV was 0.12%. The geographic prevalence rates were as follows: north (0.24%), northeast (0.15%), central-west (0.13%), southeast (0.07%), and south (0.10%). The proportion of conscripts who reported having sex with other men was 4.4%, and the estimated HIV prevalence in this group was 1.32%. Regarding prophylaxis use, 45.6% of the participants reported consistent condom use with casual partners within the last year, and 34.5% reported condom use with steady partners within the last year. The independent factors associated with HIV infection were: MSM status (odds ratio [OR] = 14.62; *P* = .000) and having more than 10 partners over their lifetime (OR = 3.32; *P* = .028).

Our data suggest that the HIV prevalence among young men in Brazil remains stable except for the north region, and MSM continue to be associated with a high risk for HIV infection at a rate that is approximately 13-fold higher than the rate among men without a history of sex with another man. Our findings confirm the need to scale up combination HIV prevention for young men, including MSM, in Brazil.

## 1 Introduction

Currently, Brazil has a stable human immunodeficiency virus (HIV)/acquired immune deficiency syndrome (AIDS) epidemic that is concentrated in key populations, although the epidemic displays distinct regional HIV burdens and trends.^[1]^ The HIV prevalence in the general population is estimated to be 0.4% at the national level, but much higher rates are found among men who have sex with men (MSM) and drug users (DU) (10.5% and 5.9%, respectively).^[2]^ According to the 2016 Brazilian HIV/AIDS Epidemiological Report, the sex ratio has increased over time; the ratio was 1.5 in 2007 and 2.4 in 2015. Among men, the AIDS detection rate has increased over the last 10 years, especially in those aged between 15 and 19 years, 20 and 24 years, and those 60 years or older. From 2006 to 2015, the AIDS detection rate among men aged 15 to 19 years more than tripled (from 2.4 to 6.9 cases/100,000 inhabitants).^[3]^

The Brazilian regions with the greatest AIDS rates are the south and north regions. The south region has shown the highest AIDS detection rate since 2000. Although this rate has shown a slight downward trend over the last several years, it is still above the national average (national rate, 20.7 cases/100,000 inhabitants; south rate, 27.9 cases/100,000 inhabitants). The north region has shown the 2nd highest AIDS rate since 2012 and has exhibited a growth trend (14.9 cases/100,000 inhabitants in 2006 and 24.0 cases/100,000 inhabitants in 2015). The AIDS detection rate in the southeast region has decreased over the last decade, whereas the rate has remained stable in the central-west region. Globally there is lack of HIV and STI surveillance data on adolescents and young men as well as their risks.^[4]^ Obtaining detailed information about the epidemic in each region of the country, especially among young men, is necessary to inform and direct specific prevention and assistance efforts for the most affected populations.^[3]^

Since 1996, the Brazilian Department of Sexually Transmitted Infections (STI), AIDS and Viral Hepatitis (Departamento de Vigilância, Prevenção e Controle das IST, do HIV, AIDS e Hepatites Virais, Secretaria de Vigilância em Saúde, Ministério da Saúde) in collaboration with the Ministry of Defense has conducted periodic and anonymous probability sample surveys to determine the HIV prevalence and gather data on the sexual and risk behaviors among conscripts of the Brazilian army.^[5,6]^ Data from these surveys are used to monitor HIV trends in young men and are applied as a proxy to estimate the prevalence of HIV among adults in the general population. Moreover, these data are used to make projections about the HIV incidence and to help allocate resources and evaluate the effectiveness of the surveillance system for the identification of HIV cases among this population.^[1,7]^

Due to the extensive geographical and cultural diversity of the country, monitoring of the HIV prevalence in this population and the adolescent sexual risk behaviors in all Brazilian regions through the collection of data at the subnational levels is an important tool to identify areas of high prevalence of HIV and the socio-behavioral aspects that contribute to new HIV infections, and subsidize the formulation of public health policies for the prevention and control of HIV and other STIs.

This article aims to present the HIV prevalence estimate and sexual risk behavior results of the 8th edition of the Conscript Survey performed in 2016 by geographic region.

## 2 Methods

### 2.1 Subject selection and sampling

The cross-sectional study was conducted with young men across Brazil aged 17 to 22 years who were in compulsory military service from August to December 2016.

For the sample size calculation, the prevalence of HIV infections among young men in 2007 was estimated to be 0.12%^[5]^ considering a 95% confidence interval and a bicaudal error of 0.04%. A total of 39,996 conscripts were selected to participate in the survey by following a sampling plan based on stratification in 2 selection stages. During the 1st stage, the selection commissions (SCs) were stratified by their geographical macro regions and selected with a probability proportional to size, which was defined by the frequency of conscripts estimated to participate in 2014. This sample represented 6.2% of the total number of young men required to enroll for military service throughout the country. A total of 87 SCs representing all Brazilian states were recruited as conscripts for this study. During the 2nd stage, the number of conscripts to be recruited by each SC was determined according to the SC size (ie, the conscripts were selected in a number proportional to the size of the SC). Conscripts were excluded if they were illiterate or functionally illiterate.

### 2.2 Data collection and laboratory assays

The study participants completed a self-reported anonymous questionnaire and provided blood samples for HIV, hepatitis, and syphilis infection testing. The questionnaire contained 74 questions and included questions about sociodemographic characteristics, sexual behavior practices, problems related to STIs, and the use of licit/illicit drugs. For the purpose of this analysis, participant drug use was considered based on whether the participant indicated one of the following options: uses from time to time, uses every day, or uses almost every day. Consistent condom use was determined to occur if the participant selected “used a condom every time.” Participants were classified as MSM if they reported having sex “only with men” and “with men and women.” All questionnaires were processed at the Laboratório de Pesquisa em HIV/AIDS (Universidade de Caxias do Sul, Caxias do Sul, RS, Brazil) using OpenText TeleForm 11.1 (Waterloo, ON, Canada).

Specimens were obtained from all recruited conscripts and tested for HIV using the Elecsys HIV combi PT (Roche Diagnostics GmbH, Penzberg, Germany). Samples that were nonreactive were classified as HIV negative, and no additional tests were performed on these samples. Specimens with positive results were subjected to the RealTime HIV-1 Viral Load Assay (Abbott Molecular Inc., Des Plaines, IL). Specimens with a viral load greater than 5000 copies/mL were classified as HIV positive, whereas specimens with a viral load of 4999 copies/mL or below the assay detection limit were subjected to the New LAV Blot I Assay (Bio-Rad, Marnes-la-Coquette, France). Specimens with a positive Western blotting result were classified as positive, whereas samples with a negative Western blotting result were considered negative; samples with indeterminate results on the Western blot were excluded from the analysis. All HIV tests were conducted at a central laboratory (Vespasiano, MG, Brazil).

### 2.3 Statistical analysis

All analyses were performed with the complex samples package of SPSS Statistics, version 22.0 (IBM Corp, Armonk, NY). The analyses incorporated data weighting, clustering (because SCs with different sizes were included), and stratification.

Since the dataset was obtained using a complex sample that combined stratification and conglomeration, the design of the survey was incorporated into the statistical analysis of the data. Additionally, a calibration procedure needed to be applied for the samples according to the census distribution by population size of the city of residence (less than 80,000 inhabitants, 80,000–199,999 inhabitants, and equal or greater than 200,000 inhabitants), age and education level.

Qualitative variables are presented as absolute and relative frequencies, and quantitative variables are presented as the mean and standard deviation. The HIV prevalence was expressed with a binomial confidence interval of 95%.

In the multivariate analysis, logistic regression was used to look at factors that were mostly associated with HIV infection in 2016. Initially, univariate logistic regression was used to calculate crude odds ratios (ORs). In the multivariate analysis, all variables potentially associated with HIV infection were included as follows: education level; MSM status; more than 5 casual partners within the last year; more than 10 partners throughout the lifespan to date; consistent condom use; and paid or received money for sex and at least one problem related to an STI. A stepwise procedure was used for the selection of joint variables associated with HIV, with variables included and excluded in each step based on the likelihood ratio.

## 3 Results

### 3.1 General characteristics

A total of 38,247 conscripts aged 17 to 22 years were enrolled in the study. From the enrolled, 965 were excluded (2.5%) due to lack of information regarding age, origin (municipality), and educational level. Thus, 37,282 (93.2%) young men across Brazil were considered for the analysis. Table 1 presents the general characteristics of the study population. The mean age of the participants was 18 years (standard deviation: 0.8), 98.2% were single, and 93.6% lived with their parents or relatives. Regarding the educational level, 93.5% completed elementary education, 50.7% completed high school, and 67.0% were still in school. Most participants reported their mothers had completed elementary (55.8%) or high school (41.2%) education, with significantly lower educational levels reported for their fathers, (47.2%) and (33.9%) for elementary and high school, respectively.

**Table 1:**
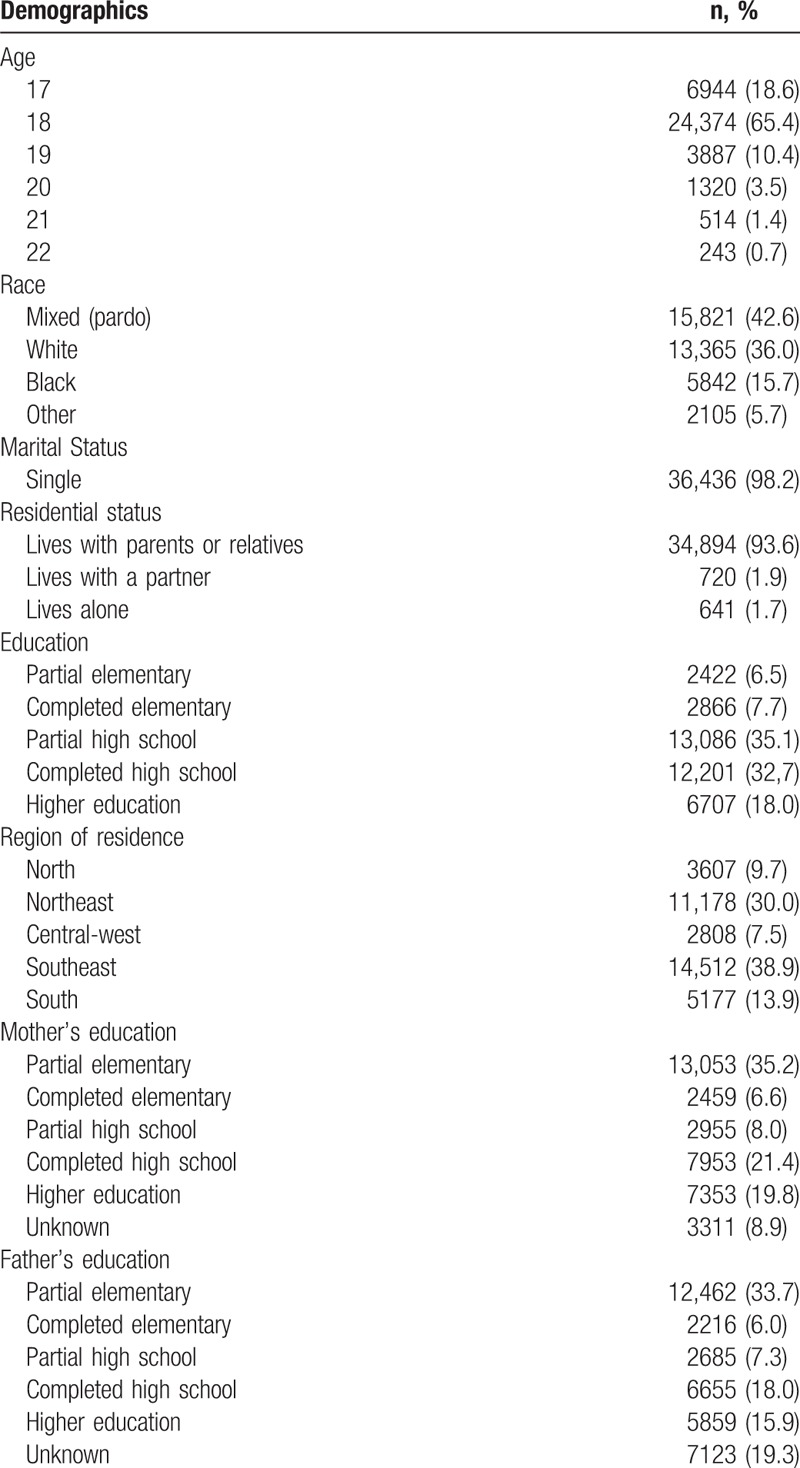
Demographic characteristics of conscripts (n = 37,282).

The majority of the population self-reported their race as mixed (pardo) (42.6%), followed by white (36.0%) and black (15.7%).

Generally, the distribution of the conscripts across regions and urban/city levels resembled that of the Brazilian population. The southeast and northeast regions contributed with the largest number of conscripts (38.9% and 30.0%, respectively), followed by the south (13.9%), north (9.7%), and central-west (7.5%) regions.

### 3.2 HIV prevalence

**Figure 1:**
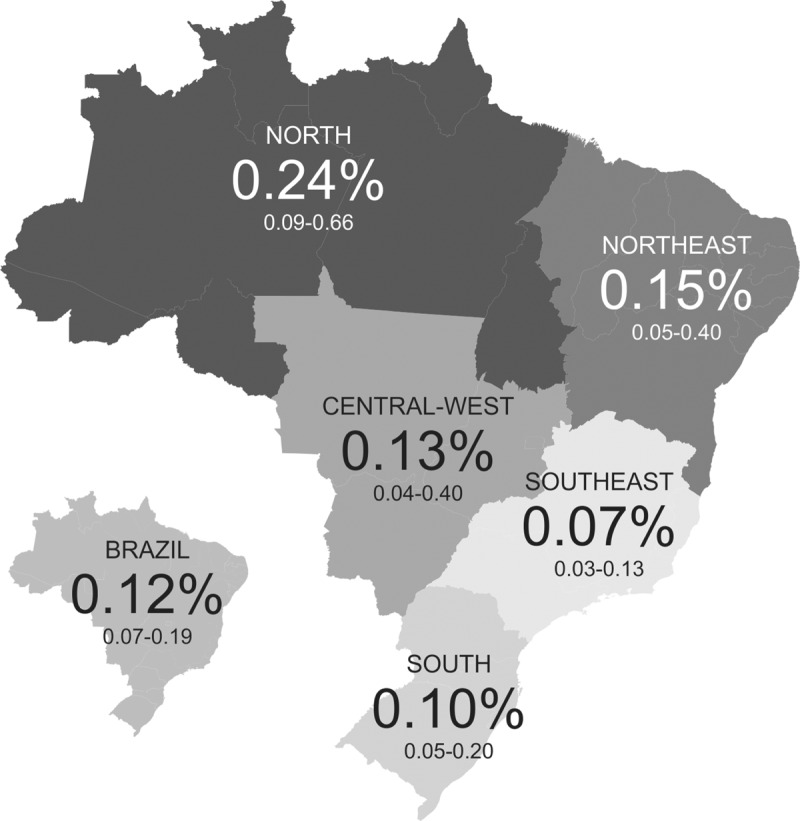
Human immunodeficiency virus (HIV) prevalence of conscripts by macro region, Brazil, 2016.

The estimated HIV prevalence rates across the country by macroregion are shown in Fig. 1. The rates ranged from 0.07 (southeast) to 0.24 (north). Notably, the HIV prevalence rate in the north region was 0.24%, whereas the HIV prevalence rate for the country as a whole was 0.12%. We found an estimated HIV prevalence rate among MSM of 1.32%; in contrast, the estimated HIV prevalence among non-MSM was 0.08% (*P* = .000) (data not shown).

Among the HIV-positive participants, the proportions of patients who tested positive for syphilis and hepatitis B infection were 12% and 19%, respectively. No patients were found with HIV-hepatitis C coinfection (data not shown).

### 3.3 Risk factors

Table 2 summarizes the sexual behavior and condom and drug use information. Among the sexually active young men (73.7%), the proportion of conscripts who had sexual relations with men (MSM) was 4.4%, whereas the proportion with more than 10 partners to date was 20.4%. Almost one-third of the sexually active conscripts had sexual intercourse before 15 years of age, and the highest percentage who reported this behavior was found in the north region. Regarding prophylaxis use, 45.6% of the participants reported consistent condom use with casual partners within the last year, and 34.5% reported condom use with steady partners within the last year. The percentage of condom use at the last sexual relation was 60.7%, and 73.8% reported condom use at the first sexual intercourse. Among the young men who had sex within the last year, the proportion of condom use when having paid sex was 69.2%, and the proportion of young men using condoms who had been paid to have sex was 63.0%. The proportion of conscripts who reported consistent use of condoms in sexual relations in the past year with a steady or a casual partner was 34.5% and 45.6%, respectively. However, only 21.9% of the MSM reported consistent condom use compared to 34.6% of the overall participants (*P* = .016).

**Table 2:**
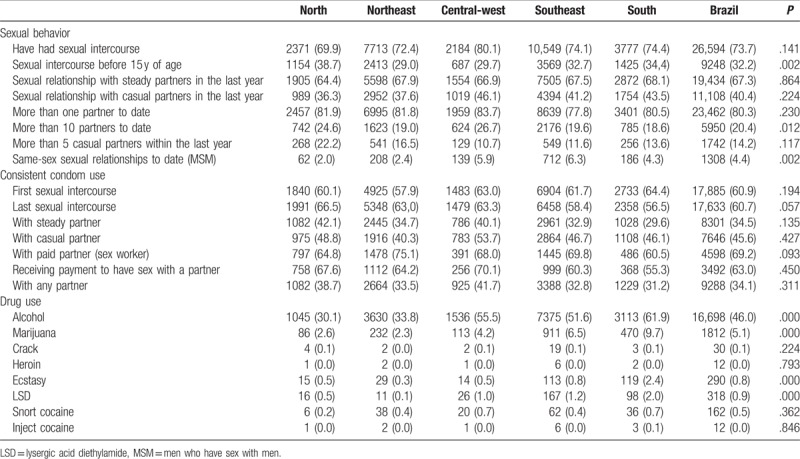
Percentages of conscripts according to sexual behavior, condom, and drug use by geographic distribution.

There was evidence of an association between the macroregion and sexual intercourse before 15 years of age (*P* = .002) in the north region (38.7%); and between the macroregion and more than 10 partners to date (*P* = .012) in the central-west (26.7%), the north regions (24.6%): and between the macroregion and same-sex relationships in life (*P* = .002) in the southeast region (6.3%).

Among the substances most often used, approximately half of the participants reported the use of alcohol (46%), followed by marijuana (5.1%), ecstasy (0.8%), lysergic acid diethylamide (LSD) (0.9%), and snorted cocaine (0.5%), with few reports of other substances. We found evidence of an association between the south region and the use of alcohol (61.9%), marijuana (9.7%), ecstasy (2.4%), and LSD (2.0%) (*P* = .000). We also found a lower proportion of consistent condom use in alcohol users (30.4%) than in nonalcohol users (37.7%) (*P* = .005).

The multivariate logistic regression results with the HIV test result as the response variable are presented in Table 3. The independent factors associated with HIV infection were: having an MSM status (OR = 14.62; *P* = .000) and having more than 10 partners over their lifetime (OR = 3.32; *P* = .028).

**Table 3:**
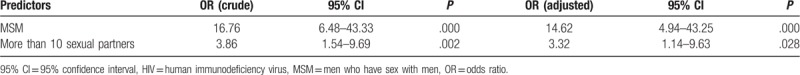
Multivariate analysis of the independent risk factors for HIV infection among conscripts in Brazil, 2016.

These results showed that although the proportion of conscripts who reported they had sex with men (MSM) was only 4.4%, the MSM were 13.6 times more likely to have HIV than their non-MSM counterparts. Additionally, having more than 10 partners over their lifetime made the conscripts 2.3 times more likely to have HIV than those with less than 10 partners.

No association was found between HIV infection and other variables, such as educational level, more than 5 casual partners within the last year, consistent condom use in those who paid or received money for sex, and at least one problem related to an STI (data not shown).

## 4 Discussion

This study was conducted as part of a series of surveys with Brazilian army male conscripts to assess HIV prevalence and associated risk factors. HIV surveillance among Brazilian army conscripts represents a unique opportunity to gather data pertaining to young men, since military service is mandatory for 18-year-old men. Thus, this group provides a long-term overview of the situation and trends of HIV prevalence and the changes in sexual and social risk behaviors associated with HIV infection. This study was conducted 10 years after the last survey (2007) and represented the largest recent HIV prevalence and risk factor study among young males in Brazil. This cohort is expected to be representative of the young men in the general population.

The estimated HIV prevalence among Brazilian army male conscripts was 0.12%, which was the same as observations in the previous edition (2007)^[5]^ and was lower than the estimated prevalence for the general population (0.4%).^[2]^ However, the estimated HIV prevalence of the north region (0.24%) was twice as high as the national average (0.12%). This result was consistent with an increase in the HIV prevalence in young men in Amazonas state, which is located in the north region, reported by Oliveira et al^[8]^ and with growth in the AIDS detection rate per 100,000 inhabitants in the general population reported for all states located in the north region in 2016.^[3]^ MSM constituted approximately 4% of our study population; however, the estimated HIV prevalence was approximately 14-fold higher than the prevalence among the non-MSM, showing the importance of obtaining data for young key populations, as it was found to be significantly higher than the general young male population of conscripts. Furthermore, an increase in the estimated HIV prevalence was found among MSM compared with the previous edition (1.23%).^[5]^ Independent predictors of HIV infection found in our study were having an MSM status, and having more than 10 partners throughout the lifespan to date, which was in accordance with the findings from the 2007 survey. Our findings add to emerging data suggesting that the HIV epidemic is largely concentrated among MSM in Brazil,^[5,9,10]^ with increases in HIV prevalence among young MSM, and the dissemination of infection into more remote areas of the country, such as the Northern region.

Our results showed that condom use at the first and most recent sexual intercourse ranged from approximately 60% to 70%, which was similar to results from the 2007 Brazilian survey^[5]^ and a similar survey performed in the United States.^[11]^ Additionally, we found that condom use in sexual relations depended on the type of partnership, with the smallest proportion invariably found for steady partners and the largest for consistent use in paying and receiving payment to have sex with a partner. These results were in agreement with other Brazilian surveys.^[5,12]^

An important finding is that more than 50% of the young men reported condomless sex with both steady and casual partners signaled the need to target prevention actions for this population. Similarly, we found that MSM reported a smaller rate of consistent condom use than non-MSM. This finding warrants attention since this population continues to have disproportionately high burdens of HIV infection in low-, middle-, and high-income countries.^[13]^

Our results indicated that almost 50% of the participants used alcohol and approximately 5.3% reported having used at least one type of the other listed substances. The south region stood out with the highest rates of substance use, which concurs with a study published in 2014 in which this region presented the highest prevalence rate of adolescents who reported they had tried alcoholic beverages (Malta, 2014). Most alcohol users (69.6%) reported inconsistent condom use during sexual intercourse, which is considered the most important risk factor for HIV acquisition and transmission. This finding is in agreement with another Brazilian study^[14]^ that verified that most participants (64.1%) did not use condoms during sexual intercourse as a consequence of previous alcohol/drug use. Compared to the previous editions of this survey and in accordance with the 2016 Brazilian Epidemiological Report, the use of injecting cocaine continued to decline, as did the use of crack (0.8% in the 2007 survey to 0.1% in this study). The LSD usage trends increased from 0.6% to 0.9% in this survey.

Considering the findings that condom use was not consistent among an important proportion of the studied population, it is crucial to draw attention to this data and recognize that youths are under greatest risk and more vulnerable to HIV infection, as well as acknowledge the related psychosocial barriers to prevention, and strongly invest in innovative approaches to reach not only the young MSMs but also the overall youth population within the HIV combination prevention strategy.

A potential limitation of the study was the exclusion of illiterate conscripts, who were conscripted and then dismissed from military service. The self-reported questionnaire may also have led to losses due to the inadequate responses regarding relevant information. Furthermore, although an effort was made to encourage honest responses, risk behaviors related to HIV and drug use might have been underreported due to a social desirability bias. Nevertheless, these limitations are minimized under the cross-sectional study design conducted using standardized data collection instruments and laboratory measurements with a very large sample size.

## 5 Conclusions

In conclusion, these surveys to determine the HIV prevalence and sexual and risk behaviors among conscripts of the Brazilian army represent an important step forward in ongoing efforts, and to develop a comprehensive understanding of young men's sexual behavior in Brazil, that in turn can inform and guide the design of preventive interventions. Our data suggest that the HIV prevalence among young men in Brazil remains stable except for the north region, and MSM continue to be associated with a high risk for HIV infection at a rate that is approximately 13-fold higher than the rate among men without a history of sex with another man. Our findings confirm the need to scale up combination HIV prevention for young men, including young MSM, in Brazil.

## Acknowledgments

The authors thank Brazilian Ministry of Defense and all participants. The authors also thank Dr Célia Landmann Szwarcwald and her team at FioCruz, Rio de Janeiro, RJ, Brazil for their statistical assistance.

## Author contributions

**Conceptualization:** A.S. Benzaken, G.F.M. Pereira.

**Data curation:** M.P. Paganella, S.K. Kato.

**Formal analysis:** M.P. Paganella, S.K. Kato.

**Funding acquisition:** A.S. Benzaken, G.F.M. Pereira, M.C.P.d. Oliveira, R.D. Sperhacke.

**Investigation:** G.F.M. Pereira, R.D. Sperhacke.

**Methodology:** A.C. Vanni, G.F.M. Pereira, R.D. Sperhacke.

**Project administration:** A.S. Benzaken, A.C. Vanni, G.F.M. Pereira, M.C.P.d. Oliveira, R.D. Sperhacke.

**Resources:** G.F.M. Pereira, R.D. Sperhacke.

**Software:** S.K. Kato.

**Supervision:** A.C. Vanni, S.K. Kato.

**Validation:** S.K. Kato.

**Visualization:** A.C. Vanni, M.P. Paganella.

**Writing – original draft:** A.C. Vanni, M.P. Paganella, R.D. Sperhacke, S.K. Kato.

**Writing – review & editing:** A.S. Benzaken, A.C. Vanni, G.F.M. Pereira, M.C.P.d. Oliveira, R.D. Sperhacke.

## References

[1] Pereira GFM Sabidó M Caruso A Transitioning from antenatal surveillance surveys to routine HIV testing: a turning point in the mother-to-child transmission prevention programme for HIV surveillance in Brazil. BMC Infect Dis 2017;17:469. 28679418 10.1186/s12879-017-2540-4 PMC5499045

[2] Brazilian Ministry of Health. Secretariat of Health Surveillance Department of STI Aids and Viral Hepatitis. The Brazilian Response to HIV and AIDS. Global AIDS Reponse Progress Reporting (GARPR) [Internet]. Brasília, DF: Brazilian Ministry of Health; 2015. Available from: http://www.unaids.org/sites/default/files/country/documents/BRA_narrative_report_2015.pdf. Accessed October 25, 2017

[3] Brasil. Ministério da Saúde. Secretaria de Vigilância em Saúde. Departamento de DST Aids e Hepatites Virais. Boletim Epidemiológico - HIV·AIDS. Ano V, n° 1. [Internet]. Brasília; 2016. 64p p. Available from: http://www.aids.gov.br/pt-br/pub/2016/boletim-epidemiologico-de-aids-2016. Accessed October 19, 2017

[4] Baggaley R Armstrong A Dodd Z Young key populations and HIV: a special emphasis and consideration in the new WHO Consolidated Guidelines on HIV prevention, diagnosis, treatment and care for key populations. J Int AIDS Soc 2015;18:85–8. 10.7448/IAS.18.2.19438 PMC4344541 25724509

[5] Szwarcwald CL de Andrade CLT Pascom ARP HIV-related risky practices among Brazilian young men, 2007. Cad Saude Publica (Internet) 2011; 27 (Suppl 1):S19–26. 10.1590/s0102-311x2011001300003 21503520

[6] Szwarcwald CL de Carvalho MF Barbosa Júnior A Temporal trends of HIV-related risk behavior among Brazilian military conscripts, 1997–2002. Clinics (Sao Paulo) 2005;60:367–74. 16254672 10.1590/s1807-59322005000500004

[7] UNAIDS/WHO Working Group on Global HIV/AIDS and STI Surveillance. Monitoring HIV Impact Using Population-Based Surveys. Geneva: Joint United Nations Programme on HIV/AIDS (UNAIDS); 2015. 110p. Available from: http://www.unaids.org/sites/default/files/media_asset/JC2763_PopulationBasedSurveys_en.pdf. Accessed September 14, 2017.

[8] de Oliveira RdSM Benzaken AS Saraceni V HIV/AIDS epidemic in the State of Amazonas: characteristics and trends from 2001 to 2012. Rev Soc Bras Med Trop (Internet) 2015;48 (Suppl 1):70–8. 10.1590/0037-8682-0121-2013 26061373

[9] Malta M da Silva CMFP Magnanini MM Improvement of HAART in Brazil, 1998-2008: a nationwide assessment of survival times after AIDS diagnosis among men who have sex with men. BMC Public Health 2015;15:226. 25886530 10.1186/s12889-015-1530-y PMC4369842

[10] Kerr LRFS Mota RS Kendall C HIV among MSM in a large middle-income country. AIDS 2013;27:427–35. 23291540 10.1097/QAD.0b013e32835ad504

[11] Gavin L MacKay AP Brown K Sexual and reproductive health of persons aged 10–24 years – United States, 2002–2007. Morb Mortal Wkly Rep 2009;58 (SS6):1–60. 19609250

[12] Dourado I MacCarthy S Reddy M Revisiting the use of condoms in Brazil. Rev Bras Epidemiol 2015;18 (Suppl 1):63–88. 26630299 10.1590/1809-4503201500050006

[13] Beyrer C Baral SD Collins C The global response to HIV in men who have sex with men. Lancet (Lond Engl) 2016;388:198–206. 10.1016/S0140-6736(16)30781-4 27411880

[14] Baptista CJ Dourado I de Andrade TM HIV prevalence, knowledge, attitudes, and practices among polydrug users in brazil: a biological survey using respondent driven sampling. AIDS Behav 2017;1–5. 10.1007/s10461-017-1812-8 28567550

